# Study on the influence of the mass eccentric wheelset on the wheel polygonal wear of high-speed trains

**DOI:** 10.1371/journal.pone.0348499

**Published:** 2026-05-11

**Authors:** Xiaonan Zhao, Songhua Zhao, Pingping He, Feng Yu

**Affiliations:** Ningbo University of Technology, Ningbo, Zhejiang, China; Tongji University, CHINA

## Abstract

In the paper, the finite element model of the wheelset-track system is established, and the explicit transient dynamic analysis method is used to study the dynamic response. The research results show that when the mass eccentric wheelset rolls on the rails, the eccentric mass will increase the wheel-rail contact force, and the peak changes of the vertical contact force are more than those of the lateral force peak changes. As the speed increases, the mass eccentric wheelset will increase the vertical vibration energy of the wheelset-track system and make the vibration energy in the middle-high frequency range increase significantly. When the speed *v* = 300 km/h, the corresponding vibration frequency *f* = 534.82 Hz becomes very prominent, which is close to the passing frequency of high-order wheel polygonal wear. At the same time, with the increase of the dynamic unbalance *U*, the overall vibration energy of the wheelset-track system presents an increasing trend, but it has little effect on the main frequency of unstable vibration. Finally, the larger the eccentric phase difference of the wheelset mass, the more the main frequency of unstable vibration exists in the middle-high frequency region, which is easy to cause high-order wheel polygonal wear.

## 1. Introduction

**Fig 1:**
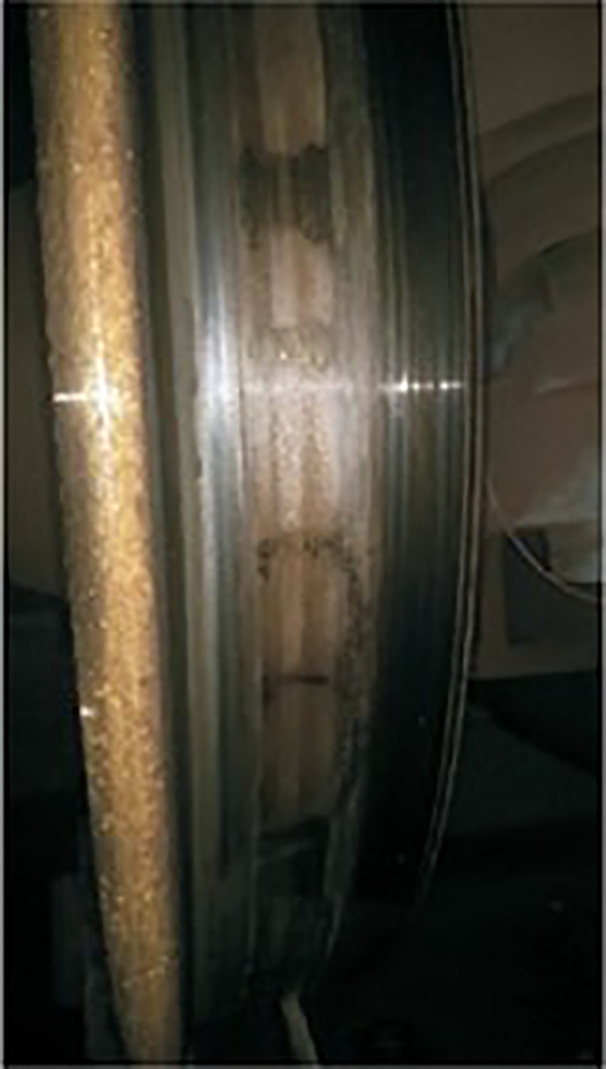
Picture of wheel polygonal wear.

With the rapid development of high-speed railways in our country, some major technical issues related to the safe operation of high-speed trains have emerged. Among them, the wheel polygonal wear is one of the most prominent technical problems [1–4], as shown in Fig 1. It can be clearly observed from Fig 1 that periodic undulations of wear have formed in the circumferential direction of the wheel tread. The wheel polygonal wear refers to the wavy wear along the circumferential direction of the tread, which will change the geometric relationship between the wheel and rail. It will produce a fixed wheel-rail contact irregularity and affect the dynamic performance of vehicles seriously. At present, there is no generally accepted formation mechanism for wheel polygonal wear. Even for the special phenomenon of wheel polygonal wear in certain trains, experts still do not have a unified view on its formation mechanism.

In recent years, experts and scholars from both domestic and foreign countries have conducted a large number of studies on the problem of wheel polygon wear. In terms of numerical simulation, Wu [5] established a refined wheel-rail contact model, using complex eigenvalue analysis and instantaneous dynamics simulation to reveal the formation mechanism of high-order polygon wear. Zhu [6] further introduced braking units and wheel hub structures, and further analyzed the influence of system parameters on wear characteristics. Dekker [7] made an analogy study between rail corrugation and wheel polygonal wear and found that the bending resonance caused the corresponding polygonal wear of the wheel. Meywerk [8] established a flexible wheelset-track model that can reproduce the development law of wheel polygonal wear, and concluded that the bending vibration frequency of wheelset is likely to be the main cause of wheel polygonal wear. Chen [9,10] constructed a finite element model of the elastic vibration of wheelset-track system. He found that the subway wheels will produce 8–13th order polygonal wear under the viscose-slip vibration condition. Chen [11] adopted the Green function method to establish a high-precision dynamic model based on the vertical interaction between track and subgrade. The numerical simulation results show that the vertical displacement and the stress of the subgrade system are mainly affected by the low-order polygonal wear of wheels. Johansson [12,13] designed the wheel polygon model by combining the track dynamics model and the wheelset wear model. The development of wheel polygonal wear can be greatly reduced by improving the isotropy of wheel material. Tao [14] concluded that there is a close relationship between the natural frequency of wheelset lateral bending and the frequency of the 24th-order wheel polygon wear. Wu [15] found that the wheel passing frequency is close to the local rail bending frequency, and the wheel polygon wear may be caused by the high-frequency vibration factor of wheel-rail contact. In terms of dynamic modeling, Ma [16] coupled the ballast track vibration model with the vehicle dynamics model to explore the initiation process of wear. Chen [17] considered the drive system and the flexibility of the wheelset, and revealed the influence of the transmission system on wear. Dong [18,19] utilized the LuGre friction model and the wheel-rail coupled rotor dynamics model to respectively investigate the role of lateral self-excited vibration and Hopf bifurcation in wear, as well as the generation and evolution of high-order polygonal wear in the wheel-rail coupled system considering the flexibility of the wheelset and the rotational effect. In terms of experiments and measurements, Huo [20] combined long-term field measurements with multi-body dynamics simulation to analyze the effect of the initial wheel-rail profile on the evolution of wear. Li [21] combined field measurement with simulation. By comparing the simulation results with the simplified and measured profiles, the necessity of considering time-varying wear in wheel-rail dynamics analysis was verified. In addition, Cai [22] systematically analyzed the effects of key parameters of the wheel-rail system on the growth and alleviation of wear.

These studies have revealed the formation mechanism and influencing factors of wheel polygonal wear from multiple perspectives, laying a solid foundation for addressing this issue. However, most current studies assume that the wheelset has an ideal mass distribution, ignoring the possible quality eccentricity problems that may exist during actual manufacturing and service. The quality eccentricity of the wheelset will generate periodic additional excitation forces on the wheelset during high-speed train operation, directly changing the contact load between the wheel and the rail and thereby exacerbating the travel and development of polygonal wear. Therefore, this paper introduces the key factor of quality eccentricity to systematically analyze its impact on the dynamic characteristics of the wheelset-track system, which has significant theoretical value and significance.

This paper addresses the problem of polygon wear of the wheel of high-speed trains, establishing a finite element model of the wheelset-track system considering quality eccentricity and using the explicit transient dynamics analysis method [23] It deeply studies the influence of quality eccentricity on the wheel-rail contact force, vibration energy distribution, and wear characteristics. The research results aim to provide a new perspective for revealing the promoting effect of dynamic imbalance caused by quality eccentricity on polygonal wear and provide theoretical basis for wheelset dynamic balance control and wear prevention.

## 2. The elastic vibration model of mass eccentric wheelset-track system

### 2.1. Mass eccentricity of the wheel

**Fig 2:**
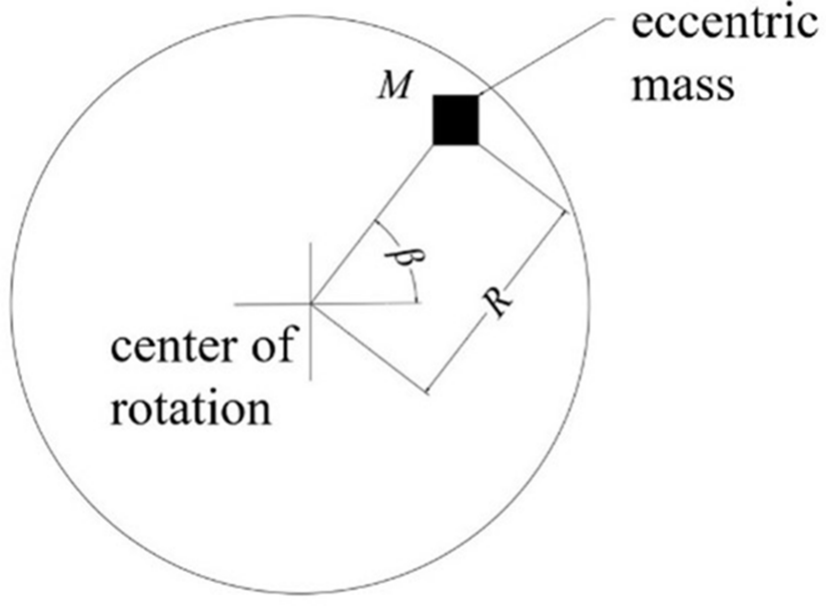
Mass eccentricity of the wheel.

**Fig 3:**
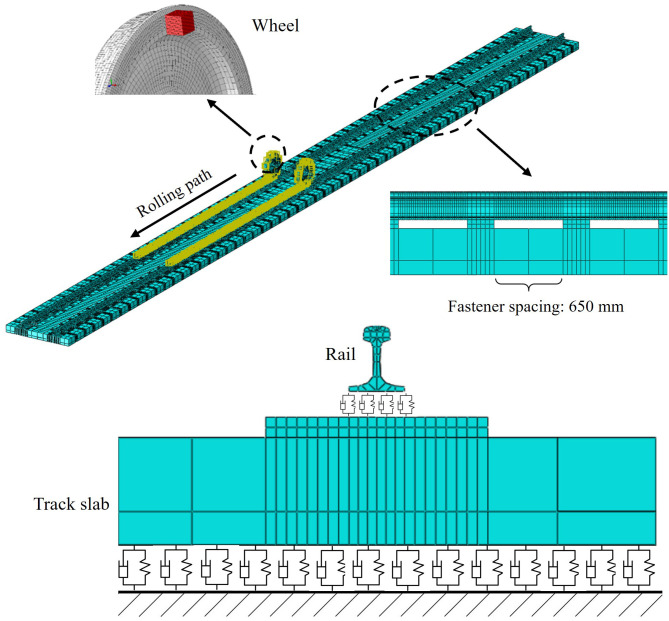
The finite element model of the wheelset-track system.


In general, the center of gravity of the wheel is located at the center of rotation. Due to machining errors in the shape of the wheel, local wear, corrosion and other reasons during operation, the center of gravity of the wheel and the center of rotation do not coincide. That is the wheel eccentricity, as shown in Fig 2. Fig 2 illustrates the geometric principle underlying mass eccentricity: the center of mass of the wheel is offset from its rotational axis by a distance R—the eccentricity. This geometric misalignment gives rise to an unbalanced centrifugal force, analytically expressed in Equation (1), which serves as the primary excitation source for all subsequent dynamic analyses. In the finite element model developed herein, the eccentricity R is physically implemented by attaching a discrete mass block to the inner surface of the wheelset, as depicted in Fig 3. The magnitude of the dynamic imbalance U is systematically varied by adjusting the material density of this block. Consequently, Fig 2 functions not merely as a conceptual schematic but as a critical conceptual and physical bridge—linking the theoretical formulation in Equation (1) with the numerical implementation—thereby establishing the foundational physical basis for investigating how operating speed, dynamic imbalance magnitude, and eccentric phase difference collectively influence the dynamic response of the wheel-rail system. At the same time, the assembly error of the axle and wheel during the press assembly process will lead to the dynamic unbalance after the wheelset assembly, which exists on all high-speed railway wheels. According to the definition of the national standard GB/T6444-1995, dynamic unbalance is the unbalanced state of the central inertia spindle of the rotating body and the rotating axis, which are both unbalanced and disjoint. The center of mass of the rotating body does not coincide with the center of rotation, and there is a distance R called eccentric distance. When the train is running, this dynamic unbalance will produce an additional moment of inertia, which will cause a change in the contact force between wheel and rail. The unbalanced centrifugal force caused in this case can be expressed as:

$$F=mR\omega^{\text{2}}$$ (1)

Where *R* is the eccentric distance, *m* is the eccentric mass, and *ω* is the angular speed of the wheel rotation. Dynamic unbalance *U* = *mR* is usually used to measure the unbalance of a wheel. In order to improve the stability of the vehicle during driving, it is necessary to minimize the dynamic unbalance of the wheel.

### 2.2. Finite element equations of motion of the wheelset-track system

When the unstable vibration of the system is studied in time domain, the nonlinear transient dynamic analysis method can be used to obtain the dynamic response. In order to fully predict the frictional self-excited vibration of the brake disc in the simulation process, Nagy [24] created a new transient dynamic analysis method, which can integrate a series of nonlinear factors in the frictional contact model. Generally speaking, the time integration method is divided into explicit integration and explicit integration, which are the transient dynamic research methods widely used. The difference between the two lies in the iterative method of acceleration solution. ABAQUS sets up solvers for two time integration methods: the former generally selected Abaqus/Explicit; the latter generally selected Abaqus/Standard. The following is a brief description of the solution steps of the explicit integration used in this paper from the theoretical level.

First, based on the explicit transient dynamic study, the dynamic response of the system is usually obtained through the diagonally concentrated mass matrix and the central difference method. Among them, when creating a stationary model of the system, it must be based on the relevant time increments, namely:


$$\text{M}{\ddot{x}}_{\left(t\right)}={\text{P}}_{\left(t\right)}-{\text{I}}_{\left(t\right)}$$ (2)

Where M is the diagonal mass matrix, ${\text{P}}_{\left(t\right)}\;$ is the applied force vector, ${\text{I}}_{\left(t\right)}\;$ is the internal force vector of the system, and subscript *t* is the time increment. Therefore, the acceleration of the system can be calculated in the following form:


$${\ddot{x}}_{\left(t\right)}={\text{M}}^{-\text{1}}{(\text{P}}_{\left(t\right)}-{\text{I}}_{\left(t\right)})$$ (3)

Then, in order to carry out explicit time integration, the central difference method is applied. The node velocity of the system at time (t+Δt) and node displacement at time $\left(t+\frac{\Delta t}{\text{2}}\right)$ can be obtained accordingly:


$${\dot{x}}_{\left(t+\frac{\Delta t}{\text{2}}\right)}={\dot{x}}_{\left(t+\frac{\Delta t}{\text{2}}\right)}+\frac{\left({\Delta t}_{\left(t+\Delta t\right)}+{\Delta t}_{\left(t\right)}\right)}{\text{2}}{\ddot{x}}_{\left(t\right)}$$ (4)

$$x_{\left(t+\Delta t\right)}=x_{\left(t\right)}+{\Delta t}_{\left(t+\Delta t\right)}{\dot{x}}_{\left(t+\frac{\Delta t}{\text{2}}\right)}$$ (5)

Where the subscripts $\left(t+\frac{\Delta t}{\text{2}}\right)$ and $\left(t-\frac{\Delta t}{\text{2}}\right)$ are the middle increments of the time increments, respectively.

The central difference method cannot be applied under the intermediate increment limit of node velocity. Therefore, the initial values of the node velocity and acceleration need to be defined. Since the system is in a stable state in the initial stage, the acceleration and velocity of the node are assumed to be 0. In the process of obtaining the next incremental step, the explicit transient dynamic analysis method does not need to analyze the convergence of the previous incremental step. So it does not need to analyze the convergence of the system during the operation. However, in order to ensure the stability of the system and achieve a certain solution accuracy, the creation of time increment step Δt must meet the following criteria. If the system is not damped, the time increment step can be constrained in the following way:


$$\Delta t\leq \frac{\text{2}}{\omega_{max}}$$ (6)

If there is damping in the system, we can control the time increment step by combining the following expressions:


$$\Delta t\leq \frac{\text{2}}{\omega_{max}}(\sqrt{\text{1}+\varepsilon^{\text{2}}-\varepsilon }$$ (7)

Where $\omega_{max}$ is the highest frequency of the system, and E represents the critical damping coefficient corresponding to the maximum of the frequency mode.

In the dynamic transient analysis, the contact formulation is the kinematic method, the contact frictional formulation is the penalty method.

To perform the dynamic transient analysis of the wheelset–track system, the steps required are as follows:

(1) Nonlinear static analysis of the wheelset–track system for applying the suspension force on the wheelset.
(2) Nonlinear dynamic analysis of the wheelset–track system for calculating the transient dynamic response.

### 2.3. Mass eccentric wheelset-track system model

**Fig 4:**
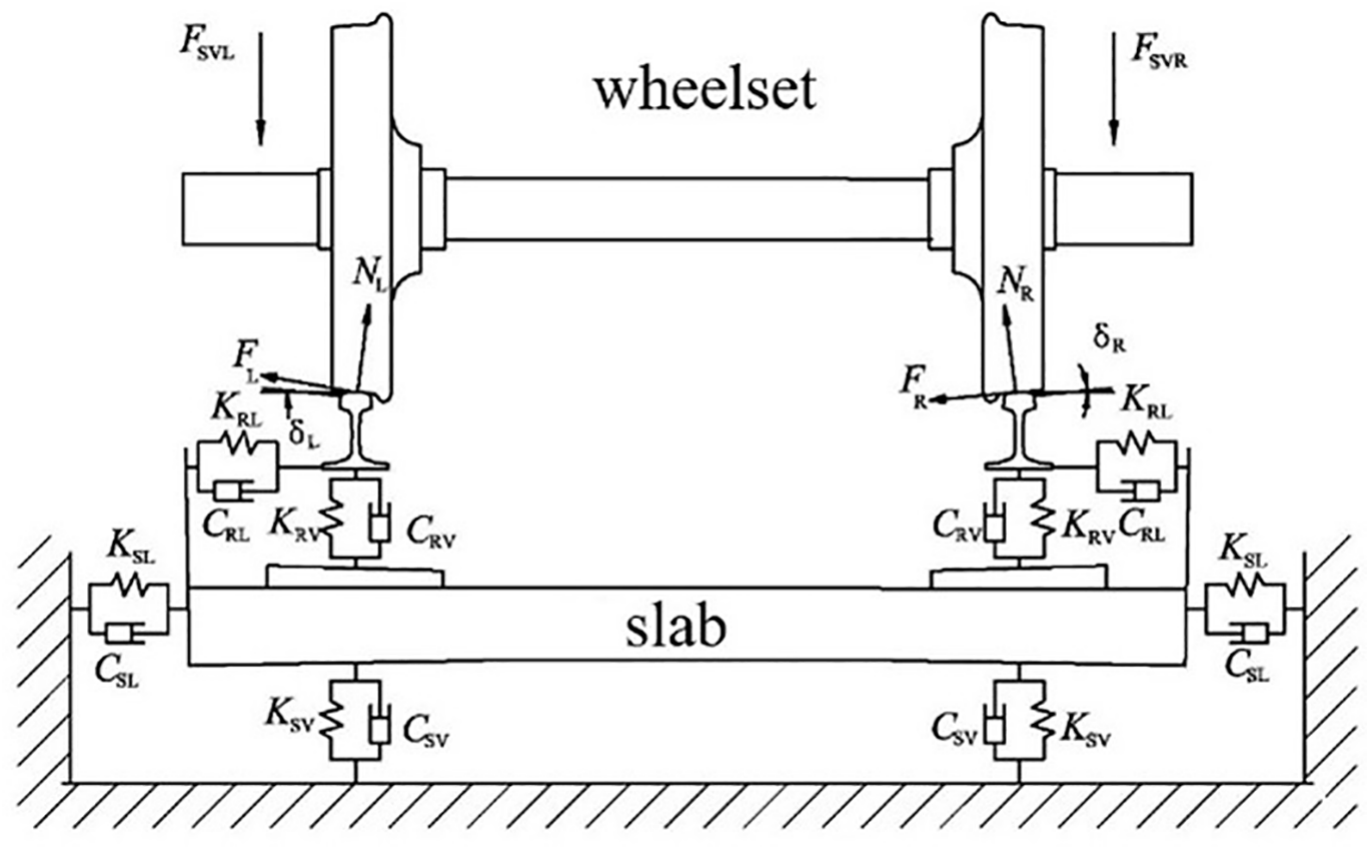
Wheel-rail contact geometry of the wheelset-track system.

In this paper, the eccentric mass block is bonded to the inner wall of the wheel. The eccentric radius *R* is unchanged, and the dynamic unbalance value *U* of the wheelset can be changed by changing the eccentric mass *m*. Fig 3 shows the details of the finite element model of the wheelset-track system. The contact positions of the left and right wheels are similar, close to the vicinity of the rail tops as shown in Fig 4.

In Fig 4, F_SVL_ and F_SVR_ are the vertical forces acting on the left and right axle boxes, respectively. δ_L_ and δ_R_ are the left and right contact angles, respectively. N_L_ and N_R_ are the normal forces at the left and right contact points, respectively. F_L_ and F_R_ are the creep forces at the left and right contact points, respectively. K_RV_ and K_RL_ are the vertical and lateral stiffness values of each fastener, respectively. C_RV_ and C_RL_ are the vertical and lateral damping values of each fastener, respectively. K_SV_ and K_SL_ represent the vertical and lateral support stiffness values from the track bed, respectively. C_SV_ and C_SL_ represent the vertical and lateral support damping values from the track bed, respectively. The rail and the sleeper are connected by the point-to-point massless spring and damper elements. The tie connection is used to connect the sleeper and the slab, and the point-to-ground massless spring and damping elements are used to simulate the track bed support for the slab. The material parameters of the components in the model are listed in  Table 1.

The calculation process is divided into two analysis steps. The first step preloads the vertical suspension force, and the second step sets the explicit transient dynamic analysis step. The analysis step size is set to 0.0001 s in the dynamic analysis. In the output of historical variables, the main parameters such as wheel-rail contact force, and the PSD analysis is carried out to obtain the main vibration frequency components of the signal. Table 1 and Table 2 give the material parameters and the track parameters of the finite element model for calculation, respectively [6,25].

**Table 1:** Material parameters of each component in the finite element model.

Parts	Poisson’s ratio	Young modulus（GPa）	Density（kg/m^3^）
Wheel	0.3	210	7.8 × 10^3^
Axle	0.29	206	7.8 × 10^3^
Rail	0.3	210	7.8 × 10^3^
Sleeper	0.2	35	2.5 × 10^3^
Slab	0.167	265	1.75 × 10^3^

**Table 2:** Finite element model parameters of track system.

Parameter	Symbol	Numerical value
Rail cant	*α*	1/40
Friction coefficient	*μ*	0.45
Sleeper pitch(mm)	*l*_s_	650
Wheel rolling circle diameter(mm)	d	920
Transverse damping of fastener (Ns/m)	*C*_RL1_	1830.22
Vertical damping of fastener (kNs/m)	*C*_RV_	20
Longitudinal damping of fastener (Ns/m)	*C*_RL2_	1830.22
Transverse stiffness of fastener(kN/mm)	*K*_RL1_	9
Vertical stiffness of fastener(kN/mm)	*K*_RV_	50
Longitudinal stiffness of fastener(kN/mm)	*K*_RL2_	9
Track plate supports lateral damping(kNs/m)	*C*_SL1_	40
Track plate support vertical damping(kNs/m)	*C*_SV_	310
Track plate supports longitudinal damping(kNs/m)	*C*_SL2_	40
Lateral stiffness of track plate support(kN/mm)	*K*_SL1_	50
Vertical stiffness of track plate support(kN/mm)	*K*_SV_	170
Longitudinal stiffness of track plate support(kN/mm)	*K*_SL2_	50
Left side vertical suspension force(N)	*F*_SVL_	60000
Right end vertical suspension force(N)	*F*_SVR_	60000

## 3. The influence of mass eccentric wheelset on the dynamics of wheelset-track system

Building upon the finite element model developed in Chapter 2, this chapter applies the explicit transient dynamic analysis method to investigate the dynamic responses of a mass-eccentric wheelset under varied operational conditions. Peak amplitudes of the wheel-rail normal contact force are systematically analyzed to quantify the influence of dynamic imbalance on contact state evolution. Furthermore, power spectral density (PSD) analysis is employed to identify dominant vibration frequencies associated with system instability across a range of parameters—including train speed, magnitude of mass eccentricity, and eccentric phase angle. These analyses collectively elucidate the intrinsic linkage between mass eccentricity characteristics and the initiation and progression of polygonal wear on the wheel tread, thereby establishing a mechanistic foundation for its formation.

### 3.1. Influence analysis of wheel-rail contact force

**Fig 5:**
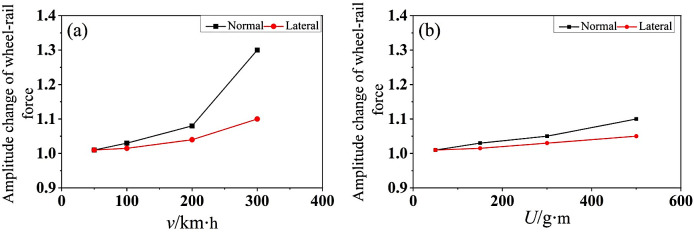
The amplitude change of wheel-rail contact force. **(a)** *U* = 150 g·m, (b) *v* = 100 km/h.

When the mass eccentric wheelset rolls on the rail, the dynamic force between the wheel and the rail will change periodically. In fact, due to the bending deformation of the wheelset, the contact relationship between the wheel and the rail will change, and the dynamic unbalance value *U* generated by the mass eccentric wheelset will have dynamic interaction on the wheel-rail contact surface both vertically and laterally. Fig 5 shows the peak variation of vertical and lateral forces between the wheel and the rail when the mass eccentric wheelset is rolled on the rail. In Fig 5(a), the dynamic unbalance value *U* = 150 g·m is kept constant. It can be seen that the peak value of wheel-rail contact force increases with the increase of speed. When *v* = 300 km/h, the corresponding peak growth multiple of wheel-rail vertical contact force is 1.3, and the peak growth multiple of wheel-rail lateral force is 1.1. In Fig 5(b), the *v* = 100 km/h remains constant. It can be seen from the Fig. that the greater the *U* value, the greater the peak value of wheel-rail contact force. When *U* = 500 g·m, the corresponding peak growth multiple of wheel-rail vertical contact force is 1.1, and the peak growth multiple of wheel-rail lateral force is 1.05. It can be seen that due to the existence of dynamic unbalance *U*, the contact state between wheel and rail becomes worse, and the change amplitude of the peak value of the vertical contact force is greater than that of the peak value of the lateral force of wheel-rail.

The vertical force variation being greater than the lateral force mainly stems from the dynamic characteristics of the system: (i) mass eccentricity-induced centrifugal forces predominantly excite vertical-mode vibrations; (ii) the system exhibits higher vertical equivalent stiffness, leading to amplified vertical displacement and force responses; and (iii) vertical energy input is more spatially and temporally concentrated, resulting in pronounced fluctuations in the normal wheel-rail contact force. According to friction-work theory, such periodic fluctuations in normal force induce corresponding oscillations in frictional work at the same frequency—thereby promoting non-uniform tread wear. Moreover, when the fluctuation frequency approaches a natural mode of the coupled wheel-rail-vehicle system, resonant amplification occurs, exacerbating wear at specific harmonic orders. Consequently, the dominance of vertical force variation constitutes a critical excitation source for subsequent frequency-domain analysis.

### 3.2. Vibration response at different speeds

**Fig 6:**
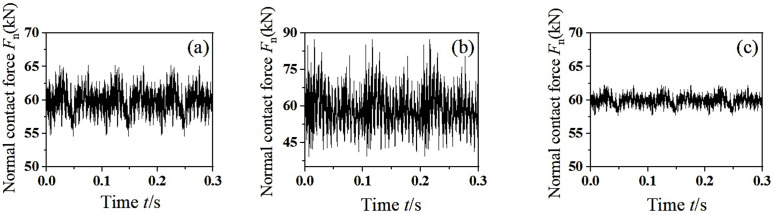
The dynamic response of normal contact force between wheel and rail. (a) *v* = 100 km/h, (b) *v* = 200 km/h, (c) *v* = 300 km/h.

According to the literature [26], the fluctuation of friction work is indirectly affected by the fluctuation of normal pressure between wheel and rail. In this section, the dynamic unbalance value *U* = 150 g·m and the mass eccentric phase difference 0° are taken as constants. Transient dynamic analysis of the finite element model of wheelset-track system is performed by applying predefined different speeds in the analysis step. The change of normal contact force between wheel and rail is extracted in the time domain. As shown in Fig 6, due to the dynamic unbalance of the wheelset, periodic unbalanced centrifugal force is generated, resulting in periodic fluctuations in the normal contact force of the wheel-rail. When the speed *v* = 100 km/h, the direction of the unbalanced centrifugal force changes periodically at any time, so the wheel-rail normal contact force fluctuates periodically near the vertical suspension force, as shown in Fig 6(a). It can be seen from Fig 6(b) and 6(c) that the fluctuation degree of wheel-rail normal contact force becomes more severe with the increase of speed. According to the view that the frictional self-excited vibration leads to wheel polygonal wear, the severe fluctuation of normal contact force will inevitably cause the fluctuation of friction work of the same frequency, and further polygonal wear will be produced on the wheel tread. In order to explore the characteristics of the wheelset-track system model in the frequency domain, the PSD analysis of the normal contact force extracted above is carried out to reveal the dominant frequency of unstable vibration of wheelset-track system. This section will use the PSD analysis toolbox in MATLAB to process the obtained time domain signal and get the corresponding frequency domain signal.

**Fig 7:**
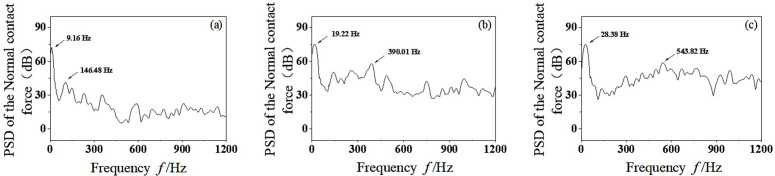
PSD analysis of normal contact force between wheel and rail. (a) *v* = 100 km/h, (b) *v* = 200 km/h, (c) *v* = 300 km/h.

According to linear vibrational theory, the fundamental frequency vibration of wheelset-track system is mainly caused by the dynamic unbalance. However, the wheelset-track system has some nonlinear factors, such as the friction between the wheel-rail and the plastic deformation of the wheelset when running on the track, which will produce other harmonic components of frequency doubling. Through PSD analysis of time domain signals, the PSD results of the wheel-rail normal contact force are obtained, as shown in Fig 7. It can be seen that the vertical vibration energy of wheelset-track system gradually increases with the increase of speed. The vibration frequencies *f* = 9.16 Hz, *f* = 19.22 Hz and *f* = 28.38 Hz correspond to the dominant frequencies of *v* = 100 km/h, *v* = 200 km/h and *v* = 300 km/h, respectively, which are the fundamental frequency vibration caused by the dynamic unbalance of the wheelset. With the increase of speed, the vertical vibration not only has higher energy at the rotating base frequency, but also increases significantly in the middle-high frequency region. When the speed reaches *v* = 200–300 km/h, the vibration energy of wheelset-track system increases obviously in the middle-high frequency region of *f* = 300–600 Hz. As can be seen from Fig 7(b) and 7(c), when *v* = 200 km/h, the main vibration frequency in the middle-high frequency region is *f* = 390.01 Hz. When *v* = 300 km/h, the corresponding main vibration frequency is *f* = 534.82 Hz. It is worth noting that when the vibration frequency corresponding to *v* = 300 km/h is *f* = 534.82 Hz, the unstable vibration frequency is likely to cause 19–20th order polygonal wear of the wheel. This is close to the vibration frequency of wheel polygonal wear detected by high-speed trains in China [27].The significant frequency arises from the resonance between the excitation frequency and the system’s natural frequency. Modal analysis of the wheelset track system reveals that a frequency of about 534.82 Hz represents the first bending mode of the wheelset. At a speed of 300 km/h, the 19th rotational frequency mode closely aligns with this bending mode, leading to resonant vibration amplification. This resonance intensifies the dynamic contact force fluctuations between the wheel and the rail, focusing frictional work on specific wavelengths and facilitating the development of high-order polygonal wear.

Therefore, it can be concluded that with the increase of speed, the mass eccentric wheelset will increase the vertical vibration energy of the wheelset-track system, which is likely to stimulate the high-order mode resonance of the wheelset and cause the wheel polygonal wear. In the process of production and operation, the dynamic unbalance of wheelset synthesis should be strictly controlled to ensure the safety, stability and comfort of vehicle operation, and reduce the occurrence of high-frequency vibration.

### 3.3. Vibration response with different dynamic unbalance value

**Fig 8:**
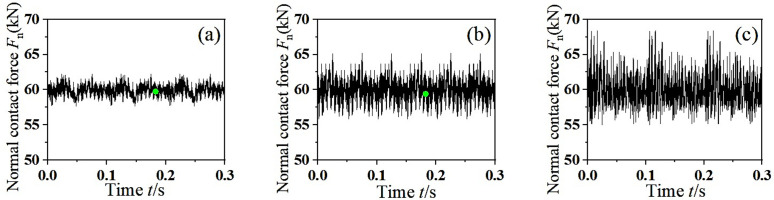
The dynamic response of normal contact force between wheel and rail. **(a)** *U* = 150 g·m, **(b)** *U* = 300 g·m, **(c)** *U* = 500 g·m.

With the abnormal wear of the wheelset in the running process, the value of the dynamic unbalance *U* will change. In this section, the influence of different dynamic unbalance on the vibration characteristics of wheelset-track system is analyzed. The speed *v* = 100 km/h and the mass eccentric phase difference 0° are taken as the fixed values. The density of mass block in the finite element model was changed to simulate different dynamic unbalance value, and the transient dynamic analysis of the wheelset-track system was carried out to extract the change of normal contact force between the wheel-rail in the time domain, as shown in Fig 8. It can be seen from the figure that the normal contact force between the wheel and rail has obvious periodic fluctuations. With the increase of dynamic unbalance, the greater the impact of centrifugal force *F* generated by high-speed rotation on wheel-rail contact surface, the more violent the fluctuation of wheel-rail normal contact force. When *U* = 500 g·m, the peak of the normal contact force between wheel and rail is close to 70 kN, which is not allowed to exist in the actual operation of the vehicle. This will not only increase the degree of wear between the wheel and the rail, but also seriously threaten the safety of driving.

**Fig 9:**
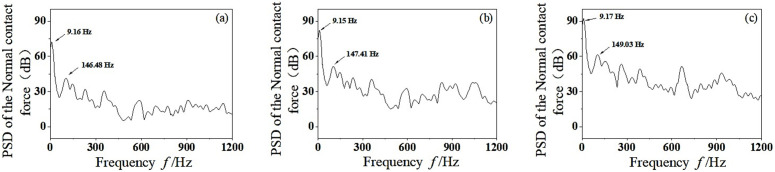
PSD analysis of normal contact force between wheel and rail. **(a)** *U* = 150 g·m, (b) *U*=300 g·m, (c) *U*=500 g·m.

PSD analysis was performed on the extracted wheel-rail normal contact force, and the dominant frequency of unstable vibration generated during wheel-rail dynamic contact was obtained, as shown in Fig 9. It can be seen that when *U* = 150 g·m, the main frequencies of normal contact force vibration between wheel and rail are *f* = 9.16 Hz and *f* = 146.48 Hz, where *f* = 9.16 Hz is the rotation fundamental frequency of the wheel. With the increase of dynamic unbalance value, the vertical vibration energy of wheelset-track system increases gradually. In addition to the fundamental frequency of rotation, the main frequency of vibration corresponding to the dynamic unbalance *U* = 300 g·m and *U* = 500 g·m is *f* = 147.41 Hz and *f* = 149.03 Hz, respectively. In conclusion, with the increase of dynamic unbalance value of the wheelset, the overall vibration energy of the wheelset-track system shows an increasing trend, but the influence on the main frequency value of the unstable vibration is little and can be ignored. This suggests that dynamic imbalance primarily affects the response amplitude rather than the resonance frequency, which depends on the system’s intrinsic stiffness and mass distribution. Therefore, controlling the degree of imbalance is crucial to reducing vibration intensity, while the sequence of polygonal wear is influenced by other factors such as speed and modal properties.

### 3.4. Vibration response caused by mass eccentric phase difference

**Fig 10:**
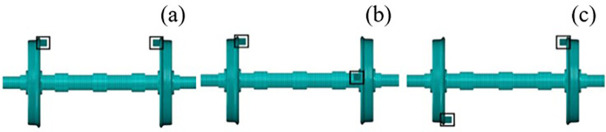
The finite element models of wheelset with different mass eccentric phase difference. (a) *α*-*β* = 0°, (b) *α*-*β* = 90°, (c) *α*-*β* = 180°.

Fig 10 shows the finite element models of wheelsets with three kinds of eccentric phase difference: (a) The eccentric mass of the left and right wheels is synchronous, that is the eccentric phase difference of the wheelset mass is *α*-*β* = 0°; (b) The eccentric mass of the left and right wheels is not synchronized and the middle value is taken, that is the eccentric phase difference is *α*-*β* = 90°; (c) The maximum is taken, that is, the eccentric phase difference is *α*-*β* = 180°. In order to discuss the influence of mass eccentric phase difference on the vibration characteristics of wheelset-track system, the speed *v* = 300 km/h and dynamic unbalance value *U* = 150 g·m are taken as the fixed values for analysis.

**Fig 11:**
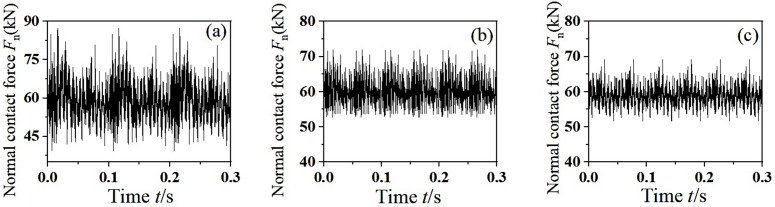
The dynamic response of normal contact force between wheel and rail. (a) *α-β*=0°, (b) *α-β*=90°, (c) *α*-*β*=180°.

Fig 11 shows the dynamic response of the normal contact force between wheel and rail. It can be seen from the figure that the normal contact force between wheel and rail fluctuates periodically near the vertical suspension force, and the peak value of the normal contact force decreases with the increasing of the eccentric phase difference of the mass. When the mass eccentric phase difference is 0°, the normal contact force of the wheel and rail fluctuates sharply, and the peak normal force reaches *F*_n_ = 80 kN. When the mass eccentric phase difference is 90°, the peak normal force decreases, and the rotating torque around the vertical line of the axle center is generated. When the mass eccentric phase difference is 180°, the wheelset swings violently around the vertical line of the center point of the axle. Due to the rotating torque around the vertical line of the axle center, the creep force between wheel and rail becomes larger and the contact condition is more severe, which is easy to cause the correlation resonance of wheelset-track system.

**Fig 12:**
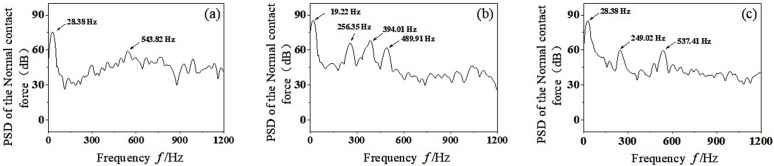
PSD analysis of normal contact force between wheel and rail. (a) *α*-*β* = 0°, (b) *α*-*β* = 90°, (c) *α*-*β* = 180°.

PSD analysis was performed on the normal contact force between wheel and rail, and the dominant frequencies of unstable vibration were obtained, as shown in Fig 12. As can be seen from the figure, with the gradual increase of the mass eccentric phase difference, the vertical vibration has a higher energy at the rotating base frequency. The main frequency of unstable vibration in the middle-high frequency region increases, which coincides with the passing frequency of the high-order wheel polygon, which may cause the wear of the high-order wheel polygon. At the same time, when the eccentric phase difference is 180°, the left and right wheels will produce longitudinal creep forces between wheels and rails in opposite directions. According to the literature [28], the opposite longitudinal creep forces will stimulate the torsion modes of the wheelset, and the torsion of the wheelset may lead to the generation of wheel polygonal wear during the operation of the vehicle. This phenomenon can be explained by the following fact: the phase difference introduces an asymmetric incentivization, which is coupled with the torsional and bending modes of the wheelset. The opposing longitudinal creep forces generate a moment about the vertical axis, thereby exciting the torsional vibration of the wheelset. This torsional vibration modulates the wheel-rail contact force at frequencies related to the torsional natural frequencies, which typically fall within the medium- to high-frequency range. This mechanism accounts for why a larger phase difference increases the number of dominant frequencies in the medium – to high-frequency region, thereby promoting high-order polygonal wear. In summary, with the gradual increase of the eccentric phase difference of the wheelset, the peak value of the normal contact force decreases. The rotating moment around the vertical line of the axle center increases, which makes the wheel-rail contact condition worse. At the same time, the main frequencies of unstable vibration in the middle-high frequency region increases, which is easy to cause the high-order polygonal wear of wheel.

## 4. Conclusions

This study employs a transient dynamic analysis method to investigate the influence of mass eccentric wheels on the dynamic response of high-speed train wheelset–track systems and their correlation with polygonal wheel wear. The principal findings are summarized as follows:

(1) The eccentricity of the mass causes an increase in the wheel-rail contact force, with the vertical force increasing more significantly than the lateral force.
(2) As the velocity increases, the vertical vibration energy of the system escalates, readily inducing higher-order modal resonance of the wheelset.
(3) An increase in dynamic unbalance value exacerbates system vibration but has minimal effect on the dominant instability frequency.
(4) An increase in the eccentric phase difference intensifies unstable vibrations in the mid- to high-frequency range, thereby increasing the risk of inducing higher-order polygonal wear.

However, this study is subject to several limitations: the model adopts an idealized mass-block representation to approximate eccentricity, thereby neglecting the dynamic evolution of eccentricity under realistic operating conditions—including thermally induced deformations, material property heterogeneity, and progressive wear accumulation. The track system is modeled using idealized boundary conditions, thereby excluding real-world complexities—including track geometric irregularities, sleeper degradation, and localized support loss; moreover, the model does not explicitly incorporate the effects of spatially varying friction coefficients, progressive wheel-rail profile wear, or the dynamic coupling among multiple wheelsets.
